# Lipids in the tumor microenvironment: immune modulation and metastasis

**DOI:** 10.3389/fonc.2024.1435480

**Published:** 2024-09-26

**Authors:** Gloria Pascual, Salvador Aznar Benitah

**Affiliations:** ^1^ Institute for Research in Biomedicine (IRB Barcelona), The Barcelona Institute of Science and Technology (BIST), Barcelona, Spain; ^2^ Catalan Institution for Research and Advanced Studies (ICREA), Barcelona, Spain

**Keywords:** tumor metabolism, lipid metabolism, tumor micreoenvironment (TME), cancer immunotherapy, metabolic adaptations

## Abstract

Tumor cells can undergo metabolic adaptations that support their growth, invasion, and metastasis, such as reprogramming lipid metabolism to meet their energy demands and to promote survival in harsh microenvironmental conditions, including hypoxia and acidification. Metabolic rewiring, and especially alterations in lipid metabolism, not only fuel tumor progression but also influence immune cell behavior within the tumor microenvironment (TME), leading to immunosuppression and immune evasion. These processes, in turn, may contribute to the metastatic spread of cancer. The diverse metabolic profiles of immune cell subsets, driven by the TME and tumor-derived signals, contribute to the complex immune landscape in tumors, affecting immune cell activation, differentiation, and effector functions. Understanding and targeting metabolic heterogeneity among immune cell subsets will be crucial for developing effective cancer immunotherapies that can overcome immune evasion mechanisms and enhance antitumor immunity.

## 1 Introduction

Metastasis is an inefficient, multistep process that accounts for over 90% of cancer deaths, yet it remains poorly understood. The metastatic process involves several sequential steps that cancer cells must undertake to enable them to spread from the primary tumor to distant sites in the body: dissemination from the primary tumor, invasion and intravasation, survival in circulation, arrest and extravasation, and colonization and growth (1).

Cancer cells interact with a variety of proteins and cells as they progress to their metastatic site. To facilitate metastasis, cancer cells must adapt to the unique microenvironment of the metastatic site, where they engage with local stromal cells to establish a supportive niche for proliferation (2, 3). These adaptive traits of cancer cells are influenced by genetic and epigenetic alterations that occur within the tumor cell itself as well as the surrounding microenvironment. In recent years, advancements in single-cell profiling and genomic analysis have shed light on the heterogeneity and evolution of metastatic tumors (4).

The tumor microenvironment (TME) is composed of tumor cells, blood vessels, extracellular matrix components, signaling molecules, and various cell types, such as immune cells, endothelial cells, pericytes, cancer-associated fibroblasts (CAFs), adipocytes, nerves, and other stromal cells. It plays a pivotal role in tumor development, progression, metastasis, and response to therapy. It also provides nutrients and survival signals, thereby influencing various aspects of cancer progression and treatment outcomes (5, 6). Recent research highlights the pivotal roles of the TME in shaping the immune response and influencing cancer progression through various mechanisms. These include promoting immune cell infiltration, facilitating communication with other cells, and secreting metabolites and immunosuppressive factors that alter immune cell function (5–7). Simultaneously, immune cells may undergo metabolic alterations due to interactions with their surroundings, leading to changes in functionality and impacting the host immune response against cancer (8). Notably, metabolic dysregulation hampers therapy efficacy and fosters immune evasion.

Thus, understanding the intricate mechanisms underlying the interactions between tumor and stromal cells and their microenvironment, which facilitate cancer metastasis, is vital for developing targeted therapies aimed at disrupting these supportive processes and impeding the growth of both primary tumors and metastases.

In this review, we thoroughly explore recent research advancements, with a specific focus on the molecular mechanisms underlying lipid metabolic rewiring in immune cells. Our aim is to investigate how these metabolic alterations influence tumor progression and metastasis, as well as their impact on the effectiveness of therapeutic interventions. Additionally, we shed light on ongoing clinical trials and novel therapeutic strategies directed towards modulating cellular immune metabolism in cancer.

## 2 Metabolic reprogramming within the TME

Metabolic reprogramming during cancer progression involves cancer and stromal cells adapting their metabolic processes to support their bioenergetic needs, thereby facilitating malignant transformation, tumor development, and cancer dissemination. Alterations in the availability, distribution, and utilization of nutrients by both tumor and stromal cells can induce this metabolic rewiring of the cells. While oncogenic mutations drive the nutritional demands of cancer cells, numerous studies have revealed metabolic reprogramming to be a pivotal factor driving tumorigenesis and cancer progression (9).

Metastatic tumors display a closer metabolic resemblance to the neighboring tissues in which they grow than to their tissue of origin. This highlights the remarkable capacity of metastatic cells to adopt characteristics reflective of their local environment, underscoring the significance of their interactions with the tissue microenvironment during metastasis (10). Emerging research suggests that metabolites in the TME can influence metastatic environments, providing metastatic tumor cells with an advantage in colonizing secondary sites more effectively due to their unique metabolic abilities (11–14). For instance, lipid-rich environments in the lung and liver contribute to the formation of pre-metastatic niches, in which the availability of palmitate and alterations in lipid metabolism play crucial roles in influencing the growth and progression of metastatic cancer cells (15). By interacting with nearby stromal cells, metastatic tumor cells can change their metabolic preferences to facilitate dissemination. In ovarian cancer, adipocytes supply fatty acids (FAs), thereby boosting the growth of distant tumors through the upregulation of the FA-binding protein FABP4 (16).

Additionally, the metabolic phenotypes of metastatic cells appear to remain dynamic and can undergo changes as the tumor progresses, leading to metabolic heterogeneity among different tumors and even within distinct regions of the same tumor (14, 15, 17). Hence, targeting a specific metabolic pathway may be effective in certain tumors or areas but not in others in the same organism due to diverse metabolic profiles, challenging the effectiveness of cancer therapy. Lee et al. found that lymph node (LN)–metastasized B16F10 melanoma tumors exhibit higher FA levels than primary tumors, with YAP activation at the LN’s invasive front sustaining FA oxidation in response to the local environment, thereby promoting metastasis (18). Notably, treatment with etomoxir, a fatty acid oxidation (FAO) inhibitor, reduces LN metastasis without affecting primary tumor size, tumor-draining LNs, or lung metastasis (19). Further, the lymph fluid environment protects metastasizing melanoma cells from ferroptosis due to having a higher level of glutathione and oleic acid and less free iron than in blood plasma. These conditions enhance the survival capacity of metastatic cells during their subsequent dissemination within the bloodstream (20). In this context, the survival and metastatic capacity of circulating tumor cells (CTCs) are helped by CTC–neutrophil clusters, allowing CTCs to evade immune surveillance and establish metastatic colonies in distant organs (21).

Immune cells within the TME also demonstrate a capacity for metabolic adaptation to the challenging tumor milieu. This metabolic reprogramming significantly influences both tumor functional characteristics and host immune responses. The interplay between cancer cells and immune cells triggers metabolic competition within the TME. In environments with low glucose levels, tumors release significant quantities of lactic acid (LA), leading to microenvironmental acidosis. In turn, regulatory T cells (Tregs) absorb LA via monocarboxylate transporter 1 (MCT1), leading to increased expression of programmed cell death protein 1 (PD-1) and thus an impairment of antitumor immunity and failure of PD-1 blockade therapy (22). Furthermore, in conditions of low glucose and acidity, tumor-infiltrating T lymphocytes (TILs) experience metabolic dysfunction and exhaustion within the TME, while macrophages are polarized towards a pro-tumoral M2 phenotype (23). Interestingly, tumor-derived exosomes drive immune–metabolic changes in lung macrophages, fostering immunosuppression through NF-κB-mediated glycolytic alterations (24).

Over the past few years, how lipid metabolism alterations affect immune cell functions within metastatic tumors has been extensively researched; we will thoroughly explore this in the upcoming sections.

### 2.1 Metabolic features of the tumor immune microenvironment

The TME plays a crucial role in driving both cancer progression and metastasis. Immune cells function to identify and eliminate abnormal cells, including cancer cells. However, as tumors advance, the interaction between cancer cells and the immune system becomes increasingly complex and dynamic, and cancer cells may develop strategies to evade immune detection and destruction, allowing them to survive and spread to distant sites in the body (25–27). During cancer progression, the immune system may become dysregulated, leading to an imbalance between pro-inflammatory and anti-inflammatory signals that creates a supportive environment for tumor growth and spread. Additionally, certain immune cells, such as Tregs and myeloid-derived suppressor cells (MDSCs), can actively suppress the immune response against the tumor, further promoting the tumor growth and metastasis (28).

Different immune cell subsets often show different metabolic characteristics, which can significantly alter their functions and behaviors within the TME. Further, when faced with challenging environments, immune cells can adjust to fulfill their energy requirements, potentially resulting in dysfunctions that subsequently influence tumoral and metastatic processes. For instance, the initial activation of T cells requires a rapid generation of energy, which is achieved by boosting aerobic glycolysis and inhibiting oxidative phosphorylation (OXPHOS) in CD8+ T cells (29). In turn, when confronted with low levels of both glucose and oxygen within the TME, CD8+ TILs heighten PPAR-α signaling and boost the degradation of FAs. This metabolic adaptation helps to sustain the effector functions of CD8+ TILs (30). On the other hand, Tregs demonstrate a selective dependency on FAO during proliferation, while being less dependent on glycolysis (31).

We increasingly understand more about how lipid metabolism and lipid signaling influence the immune system within the TME and contribute to creating an immunosuppressive milieu that promotes tumor growth and spread (32). Consequently, targeting these lipid-mediated mechanisms presents a promising and potent strategy to enhance anti-tumor immunity and to disrupt the process of metastasis (33, 34).

#### 2.1.1 Impact of dysregulated lipid metabolism on effector immune cell activity and cytotoxicity

Activated cytotoxic T lymphocytes (CTLs), together with natural killer (NK) cells, M1 macrophages, dendritic cells (DCs), and neutrophils, serve as primary effector cells in identifying and eradicating cancer cells (26, 35). Despite the challenges posed by the environment, these immune cells demonstrate adaptability, enabling them to survive within the TME and enhancing both anti-tumor and pro-tumor responses (36). CTLs are part of the adaptive immunity and rely on their varied array of clonally rearranged T-cell receptors (TCRs) to target specific peptide–MHC class I complexes on cancer cells to specifically kill them. In contrast, NK cells, which are innate lymphoid cells, utilize a spectrum of fixed activating and inhibitory receptors to regulate their activity and specificity. M1 macrophages and DCs are responsible for recognizing and then capturing cancer cells, processing their antigens, and presenting them to other immune cells, thereby activating the immune response. Neutrophils of the N1 subtype exhibit antitumor effects through both direct or antibody-dependent cytotoxicity and by enhancing the immune response via the recruitment of various immune cells (35).

Lipids serve as energy sources and metabolic components for anti-tumor immunity, particularly in enhancing the activation of CD8+ T cells, NKs, and DCs (36). Increased FA levels in the TME activate peroxisome proliferator–activated receptor (PPAR) α, supporting CD8+ T cell lipid metabolism and function (30). Boosting FA catabolism in these cells improves tumor elimination (30). PPAR-γ aids in the differentiation of effector T cells (Teffs) by enhancing FAO, while lipid depletion in CD8+ T cells leads to functional exhaustion (36) (see **Table 1**).

**Table 1:** Summary of immune modulation mechanisms discussed in the review that can be targeted for intervention, highlighting potential therapeutic targets.

Tumor Model	Tumor Location	Immune Cells	Metabolic Feature/Alteration	Effects	Tumor/Metastasis Growth	Targeted Mechanisms	Targeted Drugs
Mouse Melanoma (B16-F10)	Subcutaneous	CD8+ TILs	↓Glucose catabolism, ↑Lipid Catabolism, ↑Mitochondrial Metabolism	↑TIL survival, ↑Effector functions	**↓**	↑PPAR⍺	Fenofibrate treatment synergizes with anti-PD1 therapy
Mouse Melanoma (B16-F10), Mouse Lewis Lung Carcinoma	Subcutaneous, Lung (tail vein injected), Lung (tail vein injected)	CD8+ TILs	↑Cholesterol Biosynthesis	↑TCR Nanoclusters, ↑Enhanced T-cell Cytotoxicity	**↓**	↓ACAT1 activity, ↑LAT Phosphorylation	Avasimibe treatment synergizes with anti-PD-1 therapy
Mouse Melanoma (B16-F10), Mouse Colon Adenocarcinoma (MC38), Mouse Multiple Myeloma (Vk*MYC)	Subcutaneous, Subcutaneous, Bone marrow (tail vein injected)	CD8+ TILs	↑Lipid Uptake, ↑Arachidonic Acid Metabolism, ↑Lipid Peroxidation, ↑ROS, ↑Ferroptosis	↓Cytokine production, ↓Antitumor Activity	**↑**	↑CD36, ↑p38 MAPK	Genetic deletion of CD36 in CD8+ T cells synergizes with anti-PD-1 therapy
Mouse Melanoma (B16-F10); Mouse Colon Adenocarcinoma (MC38)	Subcutaneous	Tregs	↑Lipid Anabolism, ↑Cholesterol Anabolism, ↑GGPP Prenylation	↑TReg Activation, ↑PD-1, ↑Immunosuppression, ↓IFN-γ, ↓Inflammation	**↑**	↑SCAP/SREBP, ↑FASN, ↑HMGCR	Simvastatin, GGTI, Sr11302,T-5224 reduces PD-1 expression in vitro
Mouse Melanoma (YUMM1.7), Mouse Melanoma (Braf/PTEN)	Subcutaneous	Tregs	↑Lipid Metabolism	↑ Intratumor Treg, ↓CD8+, ↓IFN-γ, ↓TNF	**↑**	↑CD36, ↑PPARβ	Anti-CD36 mAb treatment synergizes with anti-PD-1 therapy
Mouse Mammary Carcinoma (4T1, AT3)	Mammary gland, Lung (orthotopic), Lung (tail vein injected)	NK cells	↑Lipid Storage, ↓OXPHOS	↓Antitumoral Activity	**↑**	↑Il1β	Anti-Il1β treatment improves the efficacy of adoptive transfer of mouse or human NK cells in reducing metastasis
Mouse Mammary Carcinoma (4T1, AT3, MMTV-PyMT)	Mammary gland, Lung (orthotopic)	Neutrophils	↑TG, ↓Lipolysis, ↑Lipid transfer to DTC	↑ Pro-Tumoral Activity	**↑**	↓ATGL	EIPA treatment reduces metastasis
Lung Adenocarcinoma (KPr, LL2)	Lung, Liver (tail vein injected)	Neutrophils	↑Oxidized Lipids	↑ Pro-Tumoral Activity	**↑**	↑ADRB2, ↑FGFR	Beta-blocker ICI-118,551 treatment improves survival
Mouse EL4 Lymphoma, Mouse Lewis Lung Carcinoma	Subcutaneous	PMN-MDSCs	↑TG, ↑PUFA, ↑PE, ↑PC, ↑AA, ↑PGE2	↓Intratumor CD8+, ↓Inflammation	**↑**	↑STAT5, ↑GM- CSF, ↑FATP2, ↑PTGES2	Lipofermata treatment synergizes with anti-CTLA4, anti-PD1 and anti-CSF1R therapy
Mouse Mammary Carcinoma (4T1)	Mammary gland, Lung (orthotopic)	Macrophages (LAMs)	↑Lipid and Cholesterol Metabolism Signature	↓Phagocytosis, ↑Immunosuppression	**↑**	_	_
Mouse Melanoma (B16-F10), Mouse Lewis Lung Carcinoma, Mouse Hepatoma (Hepa1-6)	Liver (intrasplenic injection)	Macrophages (MAMs)	↑Tumor-derived Lipids Uptake, ↑Lipid Accumulation (TG, CerP, PC, So, s-, mu-LCFAs), ↑Mitochondrial Respiration	↑IL4-M2-like phenotype activation, ↓GzmB ↓IFNγ of CD8+ T cells	**↑**	↑CD36	Genetic deletion of CD36 in BMDCs reduces M2 polarization and supresses liver metastasis
Mouse Epithelial Ovarian Cancer (ID8)	Peritoneal cavity (intraperitoneal injection)	Macrophages (MAMs)	↑Cholesterol Efflux	↑IL4/IL3, ↓IFNγ	**↑**	↑ABCA1/G1, ↑STAT6/PI3K-mTORC2-Akt	Anti-IL4rα mab treatment and myeloid-specific deletion of ABCA1 and ABCG1 reduces tumor growth

The ↑ arrow indicates accumulation, increased tumor, metastatic growth, and activation of various molecular pathways or components in tumors.

Conversely, the ↓ arrow indicates a decrease, reduction, or inhibition of the activity of the specified pathways or components.

Cholesterol metabolism plays a crucial role in T cell activation, and particularly in TCR clustering and signaling, which strengthens their activation (37). Research studies have revealed that cholesterol biosynthesis and transport pathways are upregulated in activated CD8+ T cells, while the cholesterol efflux pathway is downregulated (37). In mouse models of metastatic melanoma and Lewis lung carcinoma, inhibiting cholesterol esterification via genetic ablation or pharmacological inhibition of acetyl-CoA acetyltransferase 1 (ACAT1), a key cholesterol esterification enzyme, boosts effector function and proliferation specifically in CD8+ T cells, thereby enhancing their antitumor activity. Conversely, inhibiting cholesterol biosynthesis or transport reduces granule and cytokine production in CD8+ T cells. Notably, using a combined therapy of avasimibe, an ACAT inhibitor, with the anti–PD-1 monoclonal antibody (mAb) demonstrates superior efficacy over monotherapies in inhibiting tumor progression and improving survival rates (37) (see **Table 1**).

Although lipids can help to activate T cells to become effector cells, research has also shown that highly proliferating tumor cells contribute to the formation of a TME marked by glucose depletion, acidic conditions, and lipid abundance; this may not only support tumor growth and dissemination but also contribute to the creation of an immunosuppressive environment (36, 38). Further, as Teff cells display elevated proliferation rates and sustained activity, their primary energy source needs to shifts towards glycolysis and aerobic respiration to fulfill ATP requirements. Thus, a lipid-rich tumor environment could compromise the anti-tumor efficacy of immune cells, potentially redirecting them towards a pro-tumoral phenotype (38).

Treg cells are involved in maintaining immune tolerance while promoting immunosuppression in the organism, helping to prevent autoimmune pathological states. Tregs may contribute to the suppression of the immune response against tumors, potentially facilitating their growth and progression (39).

Signaling through sterol-regulatory-element-binding proteins (SREBPs) and SREBP-cleavage-activating protein (SCAP) orchestrates cellular programs that regulate lipid and cholesterol synthesis, inhibitory receptor signaling, and functional maturation of Tregs, fostering an immunosuppressive environment conducive to tumor growth (40). Oncogenic alterations, such as *RHOA* mutations, are able to activate the PI3K–AKT–mTOR pathway, boosting FA synthesis and increasing free fatty acids (FFAs) in the TME. Tregs preferentially use these FAs to fuel FAO, enhancing their survival and suppressive abilities. Gastric tumors harboring the Y42 mutation exhibit elevated levels of FFAs, resulting in resistance to anti–PD-1 mAb therapy. Importantly, co-administration of a phosphoinositide 3-kinases (PI3K) inhibitor alongside anti–PD-1 blockade effectively overcomes this resistance in *RHOA* Y42-mutated tumors (41).

**Figure 1:**
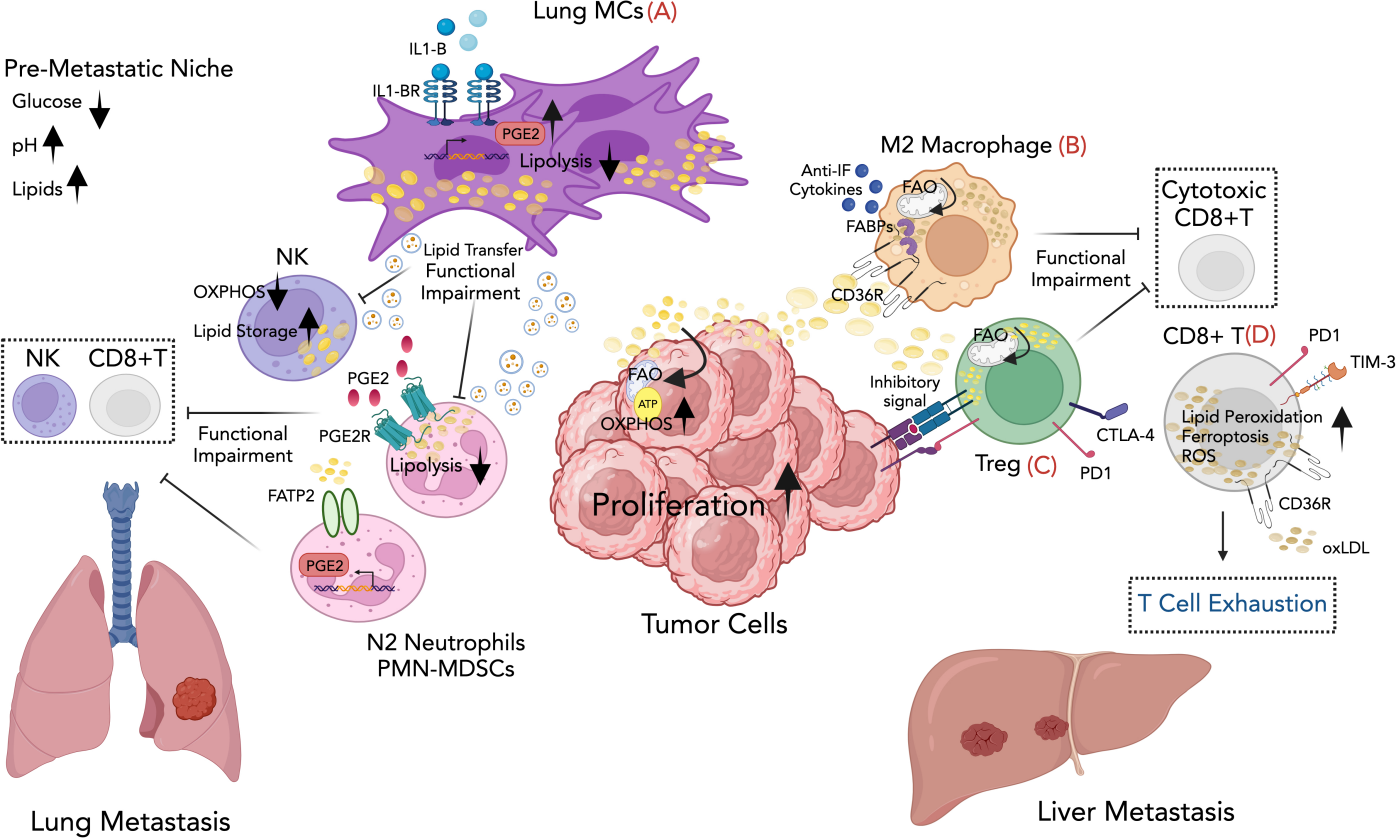
Graphical overview for some of the mechanisms involving lipid immunomodulation of metastatic growth described in the review. **(A)** Lung mesenchymal cells in the lung facilitate breast cancer metastasis by suppressing anti-tumor immunity and providing metabolic support to the metastatic tumor cells through lipid transfer to both tumor cells and NK cells via exosome-like vesicles, leading to enhanced survival and proliferation of tumor cells and dysfunction of NK cells. Lipids stored in lung neutrophils are transported to metastatic tumor cells through a macropinocytosis-lysosome pathway, endowing tumor cells with augmented survival and proliferative capacities. **(B)** Lipid-rich vesicles are selectively taken up by macrophages through CD36, providing them with fuel and activating their tumor-promoting functions. The accumulation of lipid droplets within the macrophages supports the M2 phenotype, which is characterized by the production of anti-inflammatory cytokines and the suppression of anti-tumor immune responses. **(C)** Tregs adapt metabolically to harsh TME, using fatty acids for fueling fatty acid oxidation (FAO). This adaptation enables Tregs to promote tumor progression and metastasis through immune evasion. **(D)** Fatty acid uptake by CD8+ T TILs causes lipid peroxidation and ferroptosis, weakening their anti-tumor activity and supporting cancer metastasis. Created with BioRender.com.

Tumor-derived lipids in the TME upregulate the FA receptor CD36 in Tregs and exhausted CD8+ TILs, promoting mitochondrial health and Treg adaptation to the TME (42, 43) (see **Figure 1**). Moreover, the uptake of oxidized lipids via CD36 impairs the function of CD8+ TILs, a process facilitated by lipid peroxidation and ferroptosis, which ultimately suppresses their effector functions and compromises their ability to combat tumors (43, 44). Interestingly, inhibiting CD36 in Tregs reduces melanoma growth and enhances anti–PD-1 therapy response (42) (see **Table 1**). These data suggest that targeting CD36 lipid metabolic pathways in T cells, including CD4+ and CD8+ T cells, may represent a valuable strategy to enhance anti-tumor immunity.

At the pre-metastatic stage, lung mesenchymal cells (MCs) accumulate neutral lipids, which they subsequently transport to NK cells via exosome-like vesicles. This lipid transfer results in dysfunction of NK cytotoxic cells, leading to a decreased ability to inhibit lung metastatic colonization *in vivo*. Inhibiting interleukin-1b (IL-1b) reduces lung metastasis and enhances the effectiveness of NK cell–based immunotherapy (45) (see **Figure 1**; **Table 1**).

Lipid remodeling has been identified as a specific mechanism that can protect tumor cells from immune-mediated cytotoxicity. In this context, a subpopulation of melanoma cells overexpressing the nerve growth factor receptor (NGFR) undergo changes in lipid metabolism, resulting in elevated expression of genes associated with cholesterol synthesis and FA desaturation, such as the enzymes 3-hydroxy-3-methylglutaryl-CoA reductase (HMGCR) and the fatty acid stearoyl-CoA desaturase (SCD) (46). This modification in lipid composition driven by NGFR affects the fluidity of the cell membrane, leading to a decrease in NK cell–activating ligands on melanoma cells. Consequently, the reduced cytotoxicity of NK cells against NGFR-high melanoma cells may facilitate enhanced metastasis formation. Importantly, targeting SCD is crucial for counteracting an NGFR-driven immune evasion in melanoma cells, as it normalizes the abnormal lipid composition and reverses its protective effects against NK cell surveillance (46).

#### 2.1.2 Lipid-driven, neutrophil-mediated immunosuppression in the pre-metastatic niche

Neutrophils can be recruited to the pre-metastatic niche by various chemotactic signals released from primary tumors, where they can exhibit both proinflammatory and immunosuppressive functions, thereby facilitating immune evasion by metastatic tumor cells (47–50). In the TME, neutrophils can undergo reprogramming into a state that promotes cancer progression (51). As previously mentioned, nutrient limitations within the TME trigger metabolic adaptations in immune cells that allow them to maintain their functionality. In a glucose-limited microenvironment, splenic neutrophils can maintain reactive oxygen species (ROS) production for T cell suppression through increased mitochondrial capacity and FAO. Notably, mouse 4T1 breast tumor cells are able to support this mitochondria-rich phenotype in neutrophils through their production of c-Kit ligand tumor-derived stem cell factor (SCF) (52).

Recent research has highlighted the crucial role of lung-resident mesenchymal cells in modulating neutrophil immunity via lipid signaling and metabolism, thereby facilitating the process of breast cancer lung metastasis (53). Gong et al. found that CD140a+ lung MCs express high levels of the enzyme prostaglandin-endoperoxide synthase 2 (PTGS2), leading to increased production of prostaglandin E2 (PGE2), a well-known membrane-derived lipid inflammatory signaling molecule that reprograms neutrophils to acquire an immunosuppressive phenotype. Inhibition of PTGS2, or blockade of PGE2 receptors, significantly reverses the immunosuppressive activity of lung neutrophils and mitigates lung metastasis in breast cancer models (54). In a separate study, Li et al. have shown that lung-infiltrating neutrophils undergo a process of neutral lipid accumulation upon engaging with resident MCs in the pre-metastatic lung environment. These lipids are transferred to metastatic tumor cells, prompting a metabolic shift towards lipid utilization and thereby enhancing their proliferative capacity. Mechanistically, lung MCs can inhibit the activity of adipose triglyceride lipase (ATGL) in neutrophils through both PGE2-dependent and -independent mechanisms, leading to the neutrophil lipid-laden phenotype. Pharmacological inhibition of micropinocytosis significantly reduces tumor cell colonization in the lung (55) (see **Figure 1**). In line with this, several studies have demonstrated that lipid accumulation in neutrophils profoundly impacts metastasis. For instance, in a mouse model of metastatic lung adenocarcinoma, stress-activated neutrophils secrete the proinflammatory, calcium-binding proteins of S100A8 and S100A9 that form a complex and accumulate oxidized lipids (56). Upon release, these oxidized lipids directly induce the proliferation of dormant tumor cells (DTCs) by upregulating the fibroblast growth factor receptor (FGFR) pathway. This stimulation prompts tumor cells to exit dormancy and initiate the development of new tumor lesions (56).

MDSCs belong to a heterogeneous population of mostly immature myeloid cells that can be related to the neutrophil pathway (e.g., granulocytic-MDSCs [G-MDSCs] and polymorphonuclear [PMN]-MDSCs) or to the monocyte differentiation pathway (e.g., M-MDSCs) (48, 57). MDSCs appear in response to pathological conditions like cancer, infection, or inflammation, primarily driven by dysregulated cytokine expression. These cells are typically absent under normal, homeostatic conditions. MDSCs play a crucial role in inhibiting immune responses and in fostering metastasis, by dampening the activity of T cells and other immune cells within the TME. This further contributes to cancer progression and compromises the effectiveness of cancer therapies (48, 57).

MDSCs have been the subject of extensive research. Studies have shown that the uptake of external lipids by MDSCs plays a crucial role in their metabolic and functional reprogramming, suggesting a target for intervention to enhance cancer treatment (58). The increased expression of the fatty acid transporter protein 2 (FATP2/Slc27a2) in PMN-MDSCs triggers neutrophil activation in cancer by promoting a selective and heightened uptake of arachidonic acid, leading to the subsequent synthesis of PGE2. In syngeneic sarcoma mouse models, genetic deletion or pharmacological inhibition of FATP2 hampers the capacity of PMN-MDSCs to suppress antigen-specific CD8+ T cell responses, consequently leading to a delay in tumor growth (59) (see **Figure 1**).

Lysosomal acid lipase (LAL) breaks down fats in lysosomes, aiding in digestion and recycling. It converts cholesterol esters and triglycerides to free cholesterol and FAs, thereby maintaining lipid balance. In a metastatic model of melanoma, LAL deficiency leads to LAL deficiency–induced MDSC infiltration and accumulation in organs, promoting cancer progression and metastasis via altered PPARγ-regulated gene expression and cytokine secretion (60).

Significantly, the metabolic transition in neutrophils has been confirmed in human cancer patients. For instance, elevated levels of immature neutrophils in peripheral blood have been observed in patients with ovarian cancers; these exhibit increased oxidative phosphorylation and variations in mitochondrial content as compared to healthy individuals (52).

#### 2.1.3 Influence of the lipid-rich tumor microenvironment on macrophage recruitment and polarization, and macrophage-driven immune evasion

Tumor-associated macrophages (TAMs) infiltrate tumors, influencing tumor growth, angiogenesis, immunosuppression, and tissue remodeling. In most tumors, the majority of TAMs originate from bone marrow (BM)-derived monocytic precursors, which replenish the tumor compartment, particularly during advanced disease, contributing to the progression of cancer. Their presence correlates with poor prognosis and therapy resistance (57, 61). TAMs exhibit diverse phenotypes that are influenced by signals from the TME, including classical M1-like (anti-tumor) and M2-like (pro-tumor) features, with the latter predominating in advanced cancers. TAM polarization is frequently linked to altered metabolism, with M1 macrophages favoring aerobic glycolysis, and M2 macrophages relying on oxidative metabolism (62). However, in tumor lesions, mixed phenotypes or populations with both M1 and M2 phenotypes coexist. Moreover, TAMs display considerable diversity within the TME, with various specialized subsets contributing to distinct anti- or pro-tumorigenic functions (57, 63).

Although therapeutic approaches targeting macrophages have the potential to enhance current treatments, the variability among TAMs introduces uncertainty to this strategy. Clarifying the metabolic factors controlling the dynamic changes in the heterogeneous subsets of TAMs within the tumor stroma may provide valuable insight for future therapeutic interventions (57, 64). Recent research has underscored the promise of targeting macrophage lipid metabolism as a therapeutic strategy for two key objectives: i) comprehending the heterogeneity linked with macrophage metabolic complexity, and ii) mitigating their pro-tumorigenic and pro-metastatic functions (65, 66).

Multiple studies have highlighted the importance of FA transporters and FA binding proteins (FABPs) in reprogramming macrophages in the cancer context. For instance, the adipocyte/macrophage FA-binding protein (A-FABP/FABP4) has been shown to be preferentially expressed in a subset of macrophages (CD11b+F4/80+MHCII−Ly6C−) in breast/mammary tumors, enhancing their ability to promote tumor growth and metastasis via IL-6-dependent pathways. Moreover, upregulation of A-FABP in TAMs is inversely associated with poor survival in breast cancer patients (67). Recently, Liu et al. identified a unique subset of macrophages primarily located in the tumor–adipose junctional regions of the TME in breast cancer, termed lipid-associated macrophages (LAMs). These LAMs are characterized by heightened expression of genes involved in lipid metabolism, such as FABP3, FABP4, FABP5, lipoprotein lipase (LPL), and lipase A (LIPA). Importantly, depletion of LAMs within the tumor–adipose microenvironment (TAME) synergistically enhances the anti-tumorigenic effects of anti–PD1 therapy (68).

A FA-rich environment within tumors promotes FA uptake by TAMs, resulting in the accumulation of cytoplasmic lipid droplets and the generation of lipid-laden TAMs that support an immunosuppressive phenotype (69, 70). A study using single-cell RNA sequencing (scRNA-seq) of lung macrophages from mice with mammary tumors revealed a distinct cluster resembling LAMs (71). LAMs, marked by genes like galectin 3 (*Lgals3*) and triggering receptor expressed on myeloid cells 2 (*Trem2*), are significantly elevated in tumor-bearing mice and show enrichment in lipid metabolism, extracellular matrix remodeling, and immunosuppression-related genes. Notably, LAMs are linked to resident alveolar macrophages, potentially representing an expanded subset in metastatic conditions (71).

Several studies have additionally suggested that tumor cells can release targeted signals that initiate alterations in both lipid metabolism and functional behavior of immune cells within tumor tissues. The interactions between tumor-derived secreted factors and macrophages represent an important mechanism through which tumors can shape the metastatic niche and influence metastasis. In this line, studies have investigated CD36-mediated metabolic interactions between tumor cells and macrophages in various metastatic mouse tumor models. The scavenger receptor CD36 is significantly upregulated in the subset of macrophages in the TME known as metastasis-associated macrophages (MAMs). Tumor cells release lipid-enriched vesicles that are selectively taken up by MAMs through the CD36 FA receptor. This process supplies MAMs with an energy-rich fuel source, supporting their pro-tumorigenic activities. Consequently, CD36-high MAMs adopt an immunosuppressive, M2-like phenotype, creating an environment in the TME that suppresses anti-tumor immune responses and promotes metastatic growth (see **Figure 1**). Importantly, high CD36 expression in patients with liver metastatic correlates with pro-tumoral M2-type MAM infiltration, promoting an immunosuppressive TME (72). CD36 is also expressed in other cells within the TME, where it aids in lipid absorption and fostering the immunosuppressive and pro-tumorigenic phenotype. In both humans with hepatocellular carcinoma (HCC) and in HCC mouse models, specific subsets of CAFs enriched in adipogenic pathways and characterized by CD36 expression have been identified. CD36+ CAFs uptake oxidized low-density lipoprotein (oxLDLs), which induces lipid peroxidation, activates p38 phosphorylation, and triggers secretion of the macrophage migration inhibitory factor (MIF). In turn, MIF interacts with CD74 on CD33+ MDSCs, recruiting them to the TME, thereby inhibiting effector T cell activity and promoting tumor growth (73) (see **Table 1**).

Further studies have shown that hyaluronic acid (HA), secreted by ovarian tumor cells, actively stimulates the efflux of plasma membrane cholesterol from TAMs through the ATP-binding cassette (ABC) transporters ABCA1/ABCG1. This heightened cholesterol efflux promotes the activation of IL-4 signaling in macrophages while inhibiting interferon (IFN)-gamma-induced gene expression; this leads to macrophage polarization towards a pro-tumoral phenotype, immune suppression, and ultimately, tumor progression (74).

In the early metastatic lung, alveolar macrophages loaded with tumor-derived microparticles exhibit distinct transcriptional and phenotypic profiles compared to non-loaded macrophages. Further, these macrophages demonstrate heightened mitochondrial mass and function, elevated ATP production, and increased OXPHOS, which support anti-metastatic activity. Importantly, these early alterations may represent a crucial stage in determining macrophage function during subsequent cancer metastasis (75).

Finally, TAMs can also exert a direct influence on other immune cells within the TME and influence tumor progression. In epithelial ovarian cancer (EOC), PD-L1+ TAM exosomes have been found to trigger lipid metabolic programs in CD8+ T cells, which suppresses their effector function and fostering exhaustion (76).

Overall, these studies suggest that inhibiting macrophage-mediated lipid metabolic communication with other TME components could offer a promising therapeutic avenue to disrupt the pro-metastatic activities of macrophages and enhance anti-tumor immunity. Uncovering the mechanisms behind the specialized subpopulations of macrophages that aid in facilitating various stages of the metastatic process will be crucial for the development of effective cancer therapies.

### 2.2 Dietary habits modulate immune response in metastasis

Obesity has been consistently linked to higher rates of cancer occurrence and metastasis across various types of tumors, and it currently is one of the primary preventable risk factors for cancer-related deaths (77). Obesity can influence the TME through various mechanisms, including triggering inflammation, reshaping adipose tissue, inducing insulin resistance and hyperinsulinemia, disturbing lipid metabolism, and modifying immune responses (77, 78). Understanding the intricate interplay between dietary lifestyles and cancer progression is a crucial step towards enhancing cancer treatment outcomes, particularly in individuals who are obese.

Recent studies have revealed a critical role for inflammation associated with obesity in driving cancer metastasis through the dynamic relationship among the adipose environment, immune system, and tumor cells (79, 80). In this context, secreted factors from adipose tissue influence the immune environment and subsequently affect metastatic tumor cells. For instance, interleukin 5 (IL5) is produced by adipose tissue in obesity and plays a critical role in inducing lung neutrophilia, which is exacerbated in the presence of a primary tumor. Recent research using various mouse models of metastatic luminal B breast cancer (MMTV-PyMT mice) has demonstrated that IL5 signaling directly impacts IL5rα+ monocytes, leading to their expansion and upregulation of granulocyte-macrophage colony-stimulating factor (GM-CSF; also called Csf2), thus promoting neutrophilia in both peripheral circulation and lungs. Obesity-related lung neutrophilia is associated with changes in pro-metastatic gene expression and enhanced lung metastasis in a GM-CSF dependent-manner. Remarkably, neutrophilia has been documented in obese individuals (81). Weight loss can reverse obesity-induced lung neutrophilia and reduce serum levels of GM-CSF and IL5, consequently reducing associated metastasis, in both mouse models and humans (82).

Similarly, it has been observed that leptin, an adipokine produced by adipocytes, activates the PD1/STAT3 pathway in CD8+ T effector cells, which leads to increased FAO and inhibition of glycolysis, ultimately impairing the antitumor functions of CD8+ T cells. Targeting the STAT3 function in T cells presents a potential strategy to mitigate the increased tumor burden and metastasis linked with obesity in breast cancer (83).

Other studies have highlighted the contribution of neutrophils with altered oxidative function to cancer progression within the context of obesity (79, 84). Neutrophils from obese individuals show heightened activity in genes responsible for producing ROS. Increased oxidative stress is linked to the dysfunction of lung blood vessels, thereby facilitating the movement of breast cancer cells into the lungs and promoting metastasis in MMTV-PyMT mammary tumor mice models. The mechanisms behind this involves obesity-induced changes in the secretory profile of neutrophils, which further stimulate ROS production and prompt the release of neutrophil extracellular DNA traps (NETs). NETs compromise the integrity of vascular junctions, thereby facilitating the metastatic process through transendothelial cell migration (84). Importantly, analysis of lung metastasis samples from cancer patients has showed an abundance of neutrophils with reduced levels of catalase (a powerful antioxidant enzyme), which correlates with a higher body mass index. In preclinical models, the administration of catalase, NET inhibitors, or genetic deletion of nitric oxide synthase 2 (Nos2) effectively reverses the obesity-associated breast cancer cell extravasation *in vivo* (84).

In experimental models of breast cancer, an isocaloric, high-cholesterol diet (HCD) leads to a notable increase of metastasis, thereby confirming the role of cholesterol in this process (85). This effect appears to be mediated by the actions of the cholesterol metabolite 27-hydroxycholesterol (27HC) on the function of myeloid cells, leading to elevated levels of polymorphonuclear neutrophils (PMNs) and γδ T cells and, simultaneously, reduced levels of cytotoxic CD8+ T cells both in tumors and at metastatic sites. Additionally, the inhibition or removal of cytochrome P450 CYP27A1, the enzyme responsible for the initial step in 27HC production, significantly diminishes metastasis in animal cancer models (85).

In summary, obesity contributes to chronic inflammation and immune dysfunction, impairing the ability of the body to identify and eradicate cancer cells. Moreover, obesity is linked to metabolic syndromes that aid in fueling the proliferation and dissemination of cancer cells. Therefore, prioritizing a healthy weight is vital for diminishing the likelihood of cancer and metastasis. Studying the systemic interactions between the immune system, adipose tissue, and tumors will provide insights for developing innovative cancer therapies.

## 3 Therapeutic interventions targeting lipid metabolism in immunotherapy

While immune checkpoint blockade (ICB) has achieved significant success in cancer treatment by enhancing T cell responses, its efficacy is limited among patients. Notably, metabolic reprogramming in immune and cancer cells contributes to resistance to immunotherapy, highlighting it as a potential target for intervention (33, 65, 86). Targeting lipid metabolism represents one promising approach of enhancing anti-tumor immune responses and improving the efficacy of metastatic cancer treatment. Given that lipids can exhibit both pro- and anti-metastatic properties coupled with their diverse roles in immune subsets, the effectiveness of therapies depends on understanding exactly how tumors and their associated stroma alter the FA metabolism of immune cells within the TME, along with the intricate bidirectional interactions occurring therein.

### 3.1 Targeting fatty acid uptake in immune cells enhances cancer immunotherapy

Accumulation of FAs aids in the metabolic reprogramming of immune cells, enabling them to fulfill their energy requirements and maintain functionality within the TME. Modulating lipid uptake by immune cells may present novel possibilities for disrupting tumor-promoting processes and improving the effectiveness of cancer treatments.

In liver metastasis, M2-type MAMs uptake and accumulate long-chain FAs derived from tumor cells through the upregulation of the CD36 receptor (72). FA species within cells may promote oxidative programs, enhancing FAO and mitochondrial respiration, thus driving pro-tumoral macrophage M2 polarization (87). CD36 is also upregulated in CD8+ TILs, where it facilitates the uptake of oxLDL. This process inhibits Teff functions via lipid peroxidation and ferroptosis, ultimately contributing to their exhaustion (43). The progressive accumulation of other FA receptors, such as FABPs, occurs as CD8+ TILs become more exhausted (88). In addition, CD36 controls the accumulation and the suppressive function of intratumoral Tregs within the TME (42). Genetic ablation of CD36 significantly restrains tumor growth by reducing the polarization of macrophages and limiting cytokine secretion by CD8+ TILs (43, 87). Conversely, treatment with an anti-CD36 monoclonal antibody, which interferes with CD36-mediated FA and oxLDL uptake, specifically disrupts the accumulation of intratumoral Tregs, leading to decreased tumor growth without triggering systemic autoimmunity (42). Notably, treatment with an anti-CD36 antibody has been shown to sensitize tumors to PD-1 blockade in various tumor models (42, 73). Remarkably, having a low number of CD36+ CAFs for patients with HCC is predictive of a better response to HCC immunotherapy (73) (see **Table 1**).

The induction of immunosuppressive capacities of PMN-MDSCs or lipid-laden neutrophils can also be abrogated by targeting FA uptake and trafficking. Genetic deletion of CD36 or treatment with lipofermata, a selective inhibitor of FATP2-mediated FA transport, have proven effective in diminishing tumor growth in diverse pre-clinical mouse tumor models, suggesting a promising route for enhancing current cancer immunotherapies for patients (58, 59) (see **Table 1**).

Overall, these studies have underscored the potential of targeting CD36-mediated metabolic adaptations in immune cells for disrupting the immunosuppressive TME and improving current cancer immunotherapies, as well as the possible role of CD36 as a predictive indicator of cancer treatment response. The prosaposin-derived cyclic pentapeptide, VT1021, stands out as the unique CD36-targeting agent in current cancer therapy clinical trials. It has demonstrated positive outcomes in phase I/II trials in several types of cancers, including ovarian, pancreatic, triple-negative breast cancer (TNBC), and glioblastoma (NCT03364400), and it has progressed to phase II/III for glioblastoma treatment (NCT03970447). However, instead of altering the lipid uptake functions of CD36, VT1021 triggers the TSP-1–CD36-mediated apoptotic signaling pathway, reprogramming MDSCs to produce thrombospondin-1 (TSP-1) in the TME. This induces apoptosis in tumor and endothelial cells through various mechanisms, including CD36-mediated apoptotic signaling, CD47 immune checkpoint blocking, TAM repolarization, and CTL activation and infiltration (89).

It is worth noting that ABT-510, a previously used TSP-1-mimetic, showed tolerability in phase I studies among cancer patients, both as a single treatment and when combined with chemotherapy and/or radiotherapy. However, it encountered hurdles in phase II trials due to its limited effectiveness (89).

Pharmacological inhibition of lipid transfer between neutrophils and tumor cells in the pre-metastatic niche has demonstrated efficacy in controlling breast cancer metastasis. For instance, in pre-clinical models, treating mammary tumor–bearing mice with the macropinocytosis inhibitor 5-[N-ethyl-N-isopropyl] amiloride (EIPA) notably decreases tumor cell colonization in the lungs while leaving the primary tumor unaffected (55). These findings imply that targeting lipid metabolism of the metastatic environment could be effective in controlling metastasis (see **Table 1**).

### 3.2 Disrupting lipid signaling reverses immunosuppression and reactivates effector responses

Certain lipid species, such as arachidonic acid, act as precursors for bioactive lipid mediators, such as PGE2. These mediators possess immunomodulatory properties and can enhance the suppressive activity of immune cells like MDSCs (59). In pre-clinical models, blocking PTGS2, or inhibiting the PGE2 receptors EP2 and EP4, effectively reverses the immunosuppressive activity of lung neutrophils and reduces lung metastasis in breast tumors, thus enhancing the therapeutic outcome of adoptively transferred T cells (54). Cyclooxygenase 2 (COX-2) is an enzyme which allows the release of prostaglandins. To date, numerous clinical trials have explored the efficacy of combining COX-2 inhibitors and EP4 receptor antagonists with immune checkpoint inhibitors (ICIs) across different cancer types. Overall, the effectiveness of these combination therapies is still being evaluated, and more research is needed to determine their potential roles in cancer treatment (90).

Preclinical mouse models of metastatic melanoma have demonstrated that the activation of the PPARγ pathway with a PPARγ ligand, such as 9-hydroxyoctadecadienoic acid (9-HODE), partially reverses damaged mitochondrial membrane potential and suppresses ROS overproduction in dysfunctional *Lal* ^−/−^ Ly6G^+^CD11b^+^ MDSCs cells, leading to the inhibition of MDSC-mediated stimulation of tumor cell growth and metastasis (60).

Notably, various studies present conflicting findings regarding the role of PPARγ in lung cancer. While Li H. et al. observed that PPARγ activation in myeloid cells promotes lung cancer progression and metastasis, Gou Q. et al. (2023) found that PPARγ induces PD-L1 degradation in malignant cells, potentially enhancing sensitivity to immunotherapy (91, 92). Consequently, understanding the systemic effects of PPARγ agonists on different components of the TME is crucial for effective treatments. However, recent clinical trials have highlighted the therapeutic potential of PPARγ agonists for lung cancer. Thiazolidinediones (TZDs) are pharmacological activators of PPARγ and are primarily used to treat type 2 diabetes. Notably, they have been extensively explored for use as potential anti-cancer agents against lung cancer and non-small-cell lung cancer (NSCLC) (NCT00780234; NCT00923949; NCT01342770). Moreover, TZDs demonstrate synergistic effects with conventional chemotherapy and radiotherapy. Nonetheless, clinical application of PPARγ agonists is hampered by their adverse effects, and further research and clinical trials are needed to comprehensively understand PPARγ’s actions in both tumor and stromal cells and to evaluate *in vivo* toxicity.

PI3Ks play a vital role in cancer, offering promising targets for therapy (93). PI3K signaling is deregulated in approximately 30% to 50% of human cancers, resulting in resistance to various anti-cancer therapies. PI3K inhibition not only hinders tumor growth but also alters the immune-suppressive tumor environment. To date, several inhibitors have been tested in both preclinical and clinical settings, aiming for potent efficacy with minimal toxicity to overcome therapy resistance. PI3K inhibitors synergize with ICIs, potentially enhancing antitumor immunity (41, 93). Specifically, the PI3K–AKT–mTOR pathway has been shown to stimulate FA synthesis by upregulating the expression of key enzymes involved in lipid biosynthesis. This can result in an accumulation of lipids, including FFAs, within the cell and in the TME (41). Additionally, alterations in PI3K signaling can affect lipid metabolism in immune cells, such as Tregs, which preferentially utilize FAs to fuel FAO and to enhance their survival and suppressive abilities (41). Inhibiting PI3K signaling may therefore have implications for modulating lipid metabolism in immune cells, potentially impacting their function within the TME. Further research is needed in this area to unlock the full potential of targeting PI3Ks inhibitors in cancer immunotherapy.

There is growing interest in inhibiting oxidative phosphorylation (OXPHOS) for combating tumor hypoxia, by decreasing oxygen consumption and reactivating immune response. Numerous compounds are undergoing clinical trials for their potential anticancer effects. Metformin, an inhibitor of respiratory complex I commonly used in diabetes treatment, has undergone clinical trials for various cancers, including head and neck squamous cell carcinoma (HNESCC; NCT02083692), esophageal squamous cell carcinoma (ESCC; ChiCTR-ICR- 15005940), and epithelial ovarian cancer (EOC; HUM00047900). By disrupting cellular energy metabolism, metformin activates pro-inflammatory pathways and counteracts immune suppression. In the TME, metformin exerts several effects: it reduces the density of TAMs while enhancing their ability to engulf tumor cells, promotes the anti-tumor activity of CD8+ TILs, decreases the number of Tregs and their immunosuppressive functions, and inhibits the differentiation of naïve CD4+ T cells into pro-inflammatory TH17 cells (94). In turn, the OXPHOS inhibitor phenformin has been evaluated in a phase I clinical trial for metastatic melanoma (NCT03026517). Preclinical studies suggest that phenformin may potentiate the effects of BRAF inhibitors (BRAFi) in both BRAFi-sensitive and -resistant melanoma cell lines. Additionally, phenformin can reduce MDSCs, thereby enhancing immune reactivity against melanoma.

Preclinical research has demonstrated that modifying metabolic pathways related to cholesterol metabolism could enhance the capacity of immune cells to combat tumor progression (37). Treating mice bearing tumors and metastases with avasimibe, an inhibitor of ACAT enzyme that is responsible for cholesterol esterification, results in reduced tumor growth and prolonged survival. Importantly, avasimibe has been clinically verified safe in other phase III trials targeting atherosclerosis (37) (see **Table 1**).

Additionally, statins, known for lowering cholesterol, are being explored for use in cancer treatment for their potential to impede tumor growth and influence the immune system. Studies suggest they may hinder cell growth, induce cell death, and reduce angiogenesis (95). Furthermore, statins may booster immune response by enhancing antitumor activity and dampening inflammation, possibly augmenting the effectiveness of cancer immunotherapy (96).

## 4 Concluding remarks

A comprehensive understanding of metabolic rewiring and immune cell heterogeneity within the TME is indispensable for developing precise and effective immunotherapies against cancer and metastasis. These therapies must be capable of overcoming immune evasion mechanisms while enhancing antitumor immunity.

Recent research has made significant strides in the exploration of new therapies that modulate lipid metabolism in immune cells, aiming to enhance the efficacy of cancer treatments and optimize patient outcomes. Numerous challenges still persist, however. The success of targeted or combined therapies can be impeded by heterogeneous patient populations and the absence of reliable biomarkers, for instance to identify individuals most likely to benefit from specific treatments or to assess therapy effectiveness during treatment.

Despite considerable progress in preclinical studies, our understanding of immune system dynamics in metastatic cancer patients remains incomplete, and preclinical models often fail to accurately reflect patient responses. To address this, personalized genetic and molecular profiling of tumors at the single-cell level, coupled with comprehensive assessments of each patient’s immune system, will be indispensable for enhancing responses to immune therapies and predicting treatment outcomes. Understanding the heterogeneity and functional effects of lipids and FA metabolism on each specific subset of immune cells will give us valuable insights for the development of effective novel targeted therapies.
